# Retinoic acid induced meiosis initiation in female germline stem cells by remodelling three‐dimensional chromatin structure

**DOI:** 10.1111/cpr.13242

**Published:** 2022-05-28

**Authors:** Yabin Zhang, Geng G. Tian, Xiang Wang, Changliang Hou, Xiaopeng Hu, Ji Wu

**Affiliations:** ^1^ Key Laboratory for the Genetics of Developmental & Neuropsychiatric Disorders (Ministry of Education), Bio‐X Institutes Shanghai Jiao Tong University Shanghai China; ^2^ Key Laboratory of Fertility Preservation and Maintenance of Ministry of Education Ningxia Medical University Yinchuan China

## Abstract

**Objectives:**

This study aimed to clarify the regulation and mechanism of meiotic initiation in FGSC development.

**Materials and Methods:**

FGSCs were induced to differentiate into meiosis in differentiation medium. RNA sequencing was performed to analysis the difference of transcription level. High‐through chromosome conformation capture sequencing (Hi‐C) was performed to analysis changes of three‐dimensional chromatin structure. Chromosome conformation capture further confirmed a spatial chromatin loop. ChIP‐qPCR and dual luciferase reporter were used to test the interaction between Stimulated by retinoic acid gene 8 (STRA8) protein and Trip13 promoter.

**Results:**

Compared with FGSCs, the average diameter of STRA8‐positive germ cells increased from 13 μm to 16.8 μm. Furthermore, there were 4788 differentially expressed genes between the two cell stages; Meiosis and chromatin structure‐associated terms were significantly enriched. Additionally, Hi‐C results showed that FGSCs underwent A/B compartment switching (switch rate was 29.81%), the number of topologically associating domains (TADs) increasing, the average size of TADs decreasing, and chromatin loop changes at genome region of Trip13 from undifferentiated stage to meiosis‐initiation stage. Furthermore, we validated that Trip13 promoter contacted distal enhancer to form spatial chromatin loop and STRA8 could bind Trip13 promoter to promote gene expression.

**Conclusion:**

FGSCs underwent chromatin structure remodelling from undifferentiated stage to meiosis‐initiation stage, which facilitated STRA8 binding to Trip13 promoter and promoting its expression.

## Graphical abstract

FGSCs were induced into meiosis initiation in differentiation medium (MEMα supplemented with FBS, BMP4, bFGF, RA). Meiosis‐associated genes were upregulated in Stra8‐germ cells. FGSCs underwent chromatin structure remodelling from undifferentiated stage to meiosis‐initiation stage, which facilitated STRA8 binding to the promoter of Trip13 and promoting its expression.

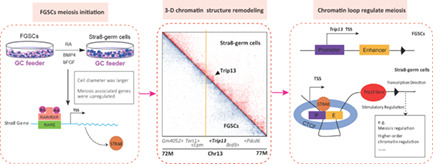

## 1 INTRODUCTION

Female germline stem cells (FGSCs) have been successfully isolated from postnatal mammalian and human ovarian tissues.^1^, ^2^, ^3^, ^4^, ^5^, ^6^, ^7^ Recent studies indicated that FGSCs transplanted into ovaries of infertile female mice in vivo had the capacity to produce normal oocytes, and fertile offsprings were produced after these mice mated with male mice.^8^ These findings suggest a new avenue for researchers to investigate the mechanism of mammalian oogenesis and treat infertility. Recently, some of studies for FGSC differentiation were reported. For example, the behaviour of FGSCs was traced after transplantation into mouse ovaries and the development process of FGSCs was characterized in vivo.^9^ In another study, human FGSCs were obtained from adult follicular aspirates and differentiated into germinal vesicle (GV)‐stage oocytes in vitro.^7^ Zou et al. compared and optimized five differentiation conditions that induced mouse FGSCs into GV‐stage oocytes in vitro.^10^ However, the underlying mechanism and regulation of early differentiation in FGSCs have remained largely unknown.

Previous studies showed that at the early stage of meiosis during gametogenesis, the expression of Stimulated by retinoic acid gene 8 (Stra8) is induced by retinoic acid (RA) and STRA8 functions as an inducer of meiotic initiation.^11^, ^12^, ^13^, ^14^, ^15^ Stra8‐deficient germ cells in females do not progress to the leptotene stage.^16^ Several reports showed that meiosis entry is defined by drastic changes in gene expression and STRA8 has transcriptional activation potential.^17^, ^18^ Kojima et al. isolated male germ cells that enter meiosis and demonstrated that STRA8, as a common transcriptional activator, directly binds the promoters of thousands of transcripts and amplifies target genes.^19^ Ishiguro et al. identified a transcription factor, MEIOSIN, which interacts with STRA8 to drive meiotic gene activation.^20^ As the sole‐known gatekeeper of the mitotic/meiotic switch in both sexes, Stra8 plays key roles in the meiotic cell‐cycle phases of germ cells, especially for premeiotic DNA replication and the subsequent meiotic events, including chromosome condensation, cohesion, synapsis and recombination.^21^ However, the underlying mechanism of meiosis initiation in FGSCs has been largely unknown.

Recently, high‐throughput chromosome conformation (Hi‐C) has been widely used in the study of three‐dimensional (3‐D) chromatin structure. Using Hi‐C technology, studies have revealed the dynamics of 3‐D chromatin organization during mammalian embryogenesis and spermatogenesis.^22^, ^23^, ^24^, ^25^, ^26^ For example, studies have shown that sperms have highly compartmentalized 3‐D chromatin organization that resembles the interphase nuclei of embryonic cells.^25^ In addition, the 3‐D chromatin structure remodelling is revealed in mouse GV oocytes and zygotes during oogenesis using single nucleus Hi‐C.^27^ During the later stage of oocytes, non‐surrounded nucleolus (NSN) oocytes transition into a surrounded nucleolus (SN) state, and this transition is important for acquiring meiotic competence.^28^ Compared with NSN oocytes, mature SN oocytes have reduced strength for topologically associating domains (TADs), compartments and loops, which are undetectable during development until metaphase II (MII) oocytes,^23^, ^29^ but TADs are reestablished after fertilization.^25^ However, the 3‐D chromatin structure of FGSCs at meiosis‐initiation stage remains to be determined.

In this study, we explored the regulation mechanism at the initiation stage of meiosis in FGSCs induced by RA in vitro. The results showed that the 3‐D chromatin structure of FGSCs was remodelled during inducing FGSC differentiation into meiosis‐initiation stage with RA. The higher‐order chromatin loop also changed at the genome region of Trip13 between FGSCs and Stra8‐positive germ cells. The STRA8 bound to the promoter of Trip13 in Stra8‐positive germ cells, which contacted distal enhancer to form spatial chromatin loop and promote its expression. These findings deepen our understanding of early in vitro differentiation in FGSCs and provided a theoretical basis for the clinical treatment of female infertility.

## 2 MATERIALS AND METHODS

### 2.1 In vitro differentiation of FGSC

Induction of FGSC differentiation was performed as described in a previous report.^10^ Briefly, STO feeder cells (derived from mouse SIM embryonic fibroblasts, strain SIM, ATCC, Manassas, VA, USA) were removed from FGSCs cultured by differential adherence. FGSCs were harvested and seeded on freshly mitotically inactivated granulosa cells (GCs) in differentiation medium containing MEMα supplemented with 5% fetal bovine serum (FBS, Life Technologies, Carlsbad, CA, USA), 10 ng/ml bFGF, 10 ng/ml bone morphogenic protein (BMP4; PeproTech, NJ, USA), 0.1 μmol/L retinoic acid (RA; Sigma‐Aldrich, St. Louis, MO, USA), 1 mM nonessential amino acids (NEAA; Invitrogen Life Sciences), 2 mM L‐glutamine (Sigma‐Aldrich), 1 mM sodium pyruvate (Amresco, Radnor, PA, USA) and 0.1 mM β‐mercaptoethanol (Sigma‐Aldrich). The medium was changed every 2 days.

### 2.2 Dual luciferase reporter assay

We plated 3 T3 cells into 24‐well plates and cultured cells until they reached 70%–80% confluence. Lipo8000 (Beyotime, Shanghai, China) was used to transfect equal amounts of pGL3‐mTrip13, pRL‐TKPCDNA3.1‐Stra8 vector for each well, together with the pGL3‐basic/pRL‐TK vector as the negative control (Sigma‐Aldrich). After 48 h, cells were harvested and analysed by the dual luciferase assay following the specific protocols (Yeasen, Shanghai, China). Each independent experiment was performed three times.

### 2.3 Chromatin immunoprecipitation (ChIP)‐qPCR

ChIP assays were performed as previously described.^30^ Briefly, the cells were cross‐linked with 1% formaldehyde for 5 min at room temperature and quenched with 125 mM glycine. Cells were then lysed with ChIP lysis buffer and sonicated to generate DNA fragments 200–1000 bp in size. The chromatin fragments were then incubated with protein A and protein G magnetic beads (1:1 mix, Sigma‐Aldrich) coupled with anti‐STRA8 (5 μg, OriGene, Rockville, MD, USA) at 4°C overnight. Antibody‐bound DNA was subsequently washed with low salt wash buffer, high salt wash buffer and LiCl wash buffer, each one time, and then TE wash buffer twice. After reverse crosslinking, the purified DNA was used for ChIP‐qPCR analysis. Primers used for ChIP‐qPCR are listed in Table S1.

### 2.4 Chromosome conformation capture (3C)

The 3C assay was performed as described previously.^31^ Briefly, 5 × 10^6^ cells were cross‐linked with 1% formaldehyde for 10 min at room temperature. Cross‐linked samples were digested overnight with 500 U of AluI (NEB, Ipswich, MA, USA) at 37°C. After inactivation, the diluted samples were ligated using T4 DNA ligase (NEB) at 16°C overnight. RNase A was then added to remove RNA for 1 h at 37°C. Proteinase K (20 mg/ml) was added to the samples at 65°C overnight. DNA was purified by phenol–chloroform extraction. The final qRT‐PCR reactions were performed in triplicate using SYBR Green. Primers for 3C‐qPCR are listed in Table S1.

### 2.5 Hi‐C library preparation and sequencing

Hi‐C libraries were generated according to the previously published protocol, with minor modifications.^32^ In brief, 2 × 10^7^ cells were harvested and re‐suspended in 45 ml of DMEM medium, followed by cross‐linking with 125 μl of 37% formaldehyde (Sigma‐Aldrich) to obtain a final concentration of 1% at room temperature for 10 min. Next, 2.5 ml of glycine was added to obtain a final concentration of 0.2 M to quench the formaldehyde. The cells were incubated for 5 min at room temperature and subsequently transferred on ice for 20 min. Fixed cells were centrifuged at 1500 rpm at 4°C for 10 min, washed with cold PBS once, and stored at −80°C. For experimental evaluation, the fixed cells were lysed with cold lysis buffer using dounce homogenizer and the subsequent steps of chromatin digestion, labelling and ligation were performed according to the protocol. Hi‐C libraries were purified using AMPure XP beads (Beckman Coulter, Brea, CA, USA). The high‐quality libraries were sequenced using an Illumina sequencing platform.

### 2.6 RNA‐seq sequencing

FGSCs were collected and total RNA was extracted by using Trizol reagent (Life Technologies) according to manufacturer's instructions. A Bioanalyzer 2100 (Agilent, Santa Clara, CA, USA) was used to assess the RNA integrity, and NanoDrop 2000 spectrophotometer (Thermo, Waltham, MA, USA) was used to evaluate RNA quantity and concentration. Sequencing libraries were created with an Illumina Hiseq2500 platform (Illumina, San Diego, CA, USA) in accordance with the manufacturer's instructions. The quality of RNA‐seq data was evaluated using FastQC. RNA‐seq reads were aligned to the reference genome (mm9) using Hisat2 (version 4.8.2).^33^ Gene expression analysis of Fragments Per Kilobase Million was calculated by Cufflinks (version 2.2.1).^34^ Sequencing depth was normalized.

### 2.7 Statistical analysis

The experiments were performed at least three times and data are shown as the mean ± SEM. Student's t‐test was used to calculate differences among groups. GraphPad Prism 6.0 software was used for performing statistical analysis. *p* < 0.05 was considered statistically significant.

## 3 RESULTS

### 3.1 Characteristics of FGSC differentiation induced in vitro

**FIGURE 1:**
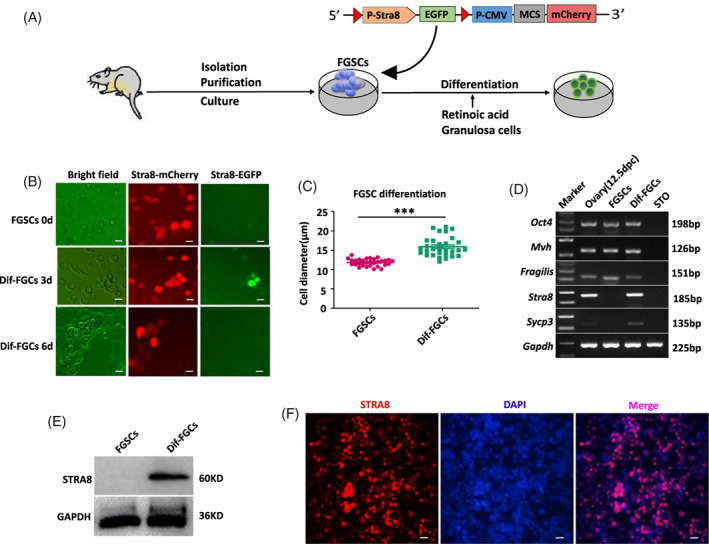
Characteristics of FGSCs differentiation induced in vitro. (A) A schematic diagram showing the culture conditions of FGSCs in vitro differentiation. (B) Representative morphology of FGSCs in the undifferentiated stage and meiosis‐initiation stage under brightfield and fluorescence microscopy. (C) Comparison of cell diameters between cells at the two stages. The cell diameter was larger for differentiated female germ cells (Dif‐FGCs) than that of FGSCs. (D) RT‐RCR analysis of female germ cell markers. Marker, 250 bp DNA marker; ovary (12.5 dpc), positive control; FGSCs, female germline stem cells; Dif‐FGCs, differentiated female germ cells at day 3; STO, negative control. (E) Western blot analysis of STRA8 and GAPDH in FGSCs and Dif‐FGCs. (F). Dif‐FGCs were detected by immunofluorescence analysis with antibodies against STRA8. Scale bars: 40 μm

To trace the early differentiation of FGSCs induced by RA in vitro, a FGSC line was identified by immunofluorescence (Figure S1) and then infected with lentivirus carrying the mCherry gene driven by the CMV promoter and the EGFP gene driven by 1.4 kb of the Stra8 promoter.^35^ When the FGSCs were exposed to differentiation Medium (MEMα supplemented with FBS, BMP4, bFGF, RA and freshly mitotically inactivated granulosa cells for feeder cells) (Figure 1A), the ratio of EGFP‐positive cells to total cells reached the highest level at day 3 and the average cell diameter increased to 16.8 μm compared with that at day 0, which was 13 μm (Figure 1B,C). Additionally, mRNA and protein levels of Stra8 were up‐regulated at day 3 (Figure 1D,E). Furthermore, immunofluorescence assay also revealed the STRA8 expression (Figure 1F). Together, these results indicated that FGSCs were induced by RA in vitro to differentiate and enter meiosis with changes in gene expression and morphology.

### 3.2 RNA‐seq revealed gene expression changes between FGSCs and Stra8‐positive germ cells

**FIGURE 2:**
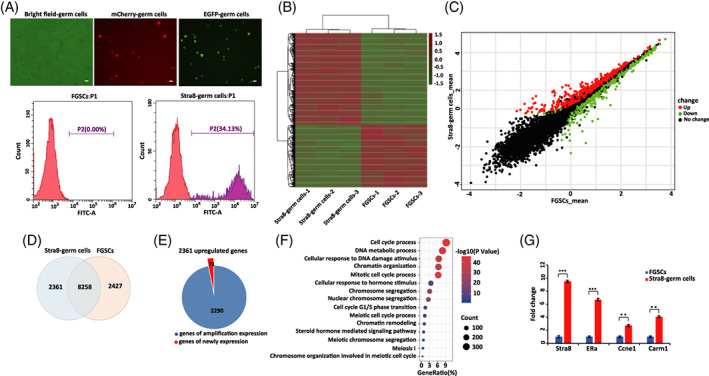
RNA‐seq revealed gene expression changes between FGSCs and Stra8‐positive germ cells. (A) Representative example of Stra8‐positive germ cell purification with Fluorescence activated Cell Sorting (FACS) and morphology of FGSCs under fluorescence microscopy after purification (upper panel) and the EGFP‐positive ratio (lower panel). Scale bars: 20 μm. (B) Hierarchical clustering showed differentially expressed genes between FGSCs and Stra8‐positive germ cells. (C) Volcano plot showed differentially expressed genes between FGSCs and Stra8‐positive germ cells. The red dots represented significantly up‐regulated genes, the green dots represented significantly down‐regulated genes and the black dots represented non‐significant differentially expressed genes. (D) Venn diagram showed the numbers of differentially expressed genes in Stra8‐positive germ cells compared with those in FGSCs. (E) Pie chart showed the number of amplified genes within the upregulated genes from the undifferentiated stage to meiosis‐initiation stage. (F) GO analysis of differentially expressed genes between FGSCs and Stra8‐positive germ cells. GO analysis was performed by DAVID. Significantly differentially expressed genes were identified as those with adjusted *p* values <0.01 and a 1.5‐fold or greater difference in expression. (G) Verification of RNA‐Seq data by qRT‐PCR. Stra8 (a meiosis‐initiation marker); ERa (a hormone associated marker); Ccne1 (a meiotic G1/S cell cycle marker); Carm1 (chromatin organization associated marker). *p < 0.05, **p < 0.01, ***p < 0.001

To investigate the changes in gene expression between FGSCs and Stra8‐positive germ cells, we isolated Stra8‐positive germ cells at day 3 using flow sorting and performed RNA‐seq. The rate of Stra8‐positive germ cells was 34.13% (Figure 2A). A total of 1426.5 million reads were generated by RNA‐seq. Hierarchical clustering of differentially expressed genes was shown by heat map in Figure 2B and volcano plot revealed the significant difference of transcripts between FGSCs and Stra8‐positive germ cells (Figure 2C). Among the 4788 differentially expressed genes, 2361 genes were up‐regulated and 2427 genes were down‐regulated (Figure 2D). Additionally, it was found that nearly 97% of the up‐regulated genes were expressed in FGSCs and 71 genes were newly expressed in Stra8‐positive germ cells (Figure 2E).

To determine the functional categories of the differentially expressed genes, we performed GO analysis and identified 2506 enriched terms. Germ cell‐associated biological processes were significantly enriched, such as meiosis‐specific processes (e.g. ‘meiosis I’, and ‘meiotic cell cycle process’), cell cycle processes (e.g. ‘cell cycle process’, ‘mitotic cell cycle’ and ‘cell cycle G1/S phase transition’) and hormone‐associated processes (e.g. ‘cellular response to hormone stimulus’ and ‘steroid hormone mediated signaling pathway’). Additionally, chromatin‐structure associated processes (e.g. ‘chromatin organization’ and ‘chromatin remodelling’) were significantly enriched (Figure 2F). Several selected genes related to the above‐enriched processes were verified by qRT‐PCR (Figure 2G). Collectively, these results indicated that the FGSCs induced by RA in vitro underwent a series of transcriptional induction events that were associated with meiosis‐specific and mitotic cell cycle processes, the hormone signal pathway and changes in chromatin structure.

### 3.3 The global comparison of chromatin structure between FGSCs and Stra8‐positive germ cells

**FIGURE 3:**
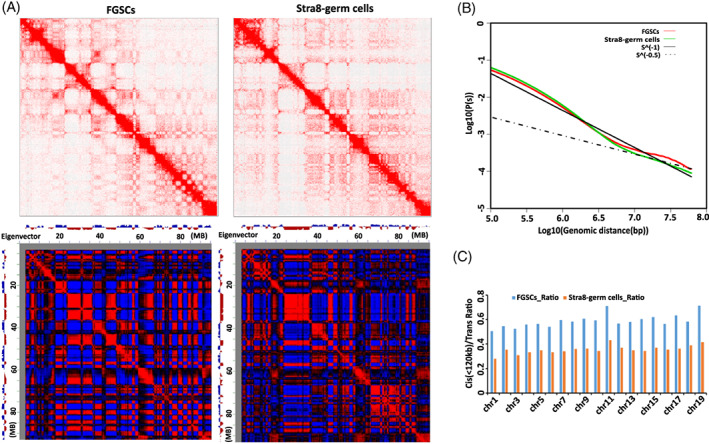
The global comparison of chromatin structure between FGSCs and Stra8‐positive germ cells. (A) The upper panel showed intrachromosomal contact matrices from chromosome 17 in FGSCs and Stra8‐positive germ cells. The colour of each dot represented the log2 of the normalized contact number for the corresponding genome bins; the lower panel showed the Pearson's correlation heat map of Chr.17 with characteristics between FGSCs and Stra8‐positive germ cells. The first principal component eigenvector identified compartment A (blue) and compartment B (red). (B) The average interaction frequency across the genome decreased with increasing genomic distance. (C) The ratio of *cis*/*trans* interactions of 19 euchromosomes in FGSCs and Stra8‐positive germ cells

To examine the differences of 3‐D chromatin structure between FGSCs and Stra8‐positive germ cells, we performed Hi‐C experiments. After filtering the invalid reads and normalization, 1.39 billion valid Hi‐C read pairs were kept as authentic chromatin contacts. Among these, 0.87 billion read pairs of long‐range (>10 kb) and 0.34 billion read pairs of short‐range (<10 kb) belong to intra‐chromosomal *cis* contacts while 0.26 billion read pairs belong to inter‐chromosomal *trans* contacts. Heat maps showed an overview of the intra‐chromosomal contact maps (chromosome 17) randomly selected and suggested that the FGSCs exhibited a distinct chromatin organization from the undifferentiated stage to meiosis‐initiation stage (Figure 3A, upper panel). Furthermore, we performed Pearson's correlation analysis of the Hi‐C matrix in chromosome 17 by extracting the eigenvectors of the whole chromosome interactions and using the first principal component (PC1) score to compare the chromosome structure in FGSCs with that in the Stra8‐positive germ cells. As shown in Figure 3A (lower panel), FGSCs and the Stra8‐positive germ cells had a strong correlation. We then examined the average intra‐chromosomal contact probability of FGSCs and Stra8‐positive germ cells. As shown in Figure 3B, the chromatin interaction frequency monotonically decreased with increasing genomic distance from 10^5^ to 10^8^ bp for both cell stages of cells. The contact probability curves were similar from 10^5^ to 10^6.5^ bp, but changes occurred over long distance in the genome.

Next, we investigated the genome‐wide chromatin interactions by calculating the *cis/trans* ratio of FGSCs and Stra8‐positive germ cells, and we found a markedly smaller ratio for Stra8‐positive germ cells compared with that in FGSCs (Figure 3C). We also observed few different observed/expected numbers of contacts of 19 euchromosomes between Stra8‐positive germ cells and FGSCs (Figure S2). These results indicated that the chromatin structure of Stra8‐positive germ cells was altered compared with that of cells in the undifferentiated stage.

### 3.4 A/B compartment changes in FGSCs from the undifferentiated stage to meiosis‐initiation stage

**FIGURE 4:**
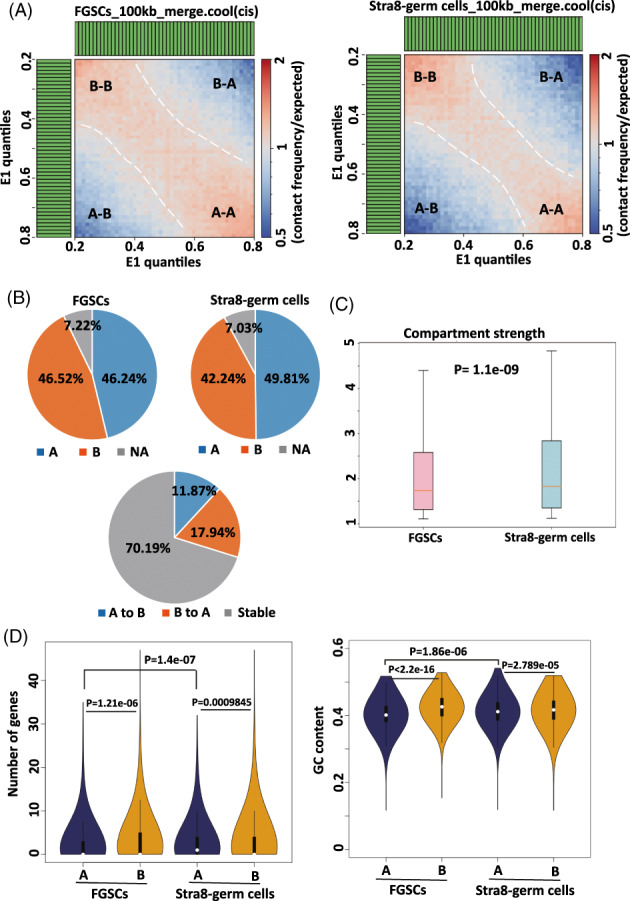
A/B compartment changes between FGSCs and Stra8‐positive germ cells. (A) The average contact enrichment arranged by PC1 value of cells at the two stages. (B) The upper panel showed the proportion of compartment A (blue) or B (orange) to the genome in each of the two stages; the lower panel showed the compartment A/B switch rate changes from FGSCs to Stra8‐positive germ cells. (C) Box plots showed the compartmentalization strength calculated for each chromosome of the two stages. (D) Violin plot descriptions of the number of genes and GC content in compartment A and B

We next systematically analysed the compartment status in the two stages of cells. On the basis of the PC1 values, we divided the genome into compartment A and B. The contact enrichment in compartment A‐A was similar to that of compartment B‐B in FGSCs (Figure 4A). However, the contact enrichment in compartment A‐A of Stra8‐positive germ cells was less than that of compartment B‐B (Figure 4A). Next, we analysed the proportion of compartment A and B genomes in the two stages of cells. Although the genome of FGSCs was almost equally divided into two parts on compartments A and B, Stra8‐positive germ cells showed marked spatial plasticity in the rearrangement of compartment A and B (Figure 4B). The switch rate of A/B compartments was 29.81% (17.94% of compartment B to compartment A and 11.87% of compartment A to compartment B), indicating that the chromatin compaction was gradually opening, allowing access for the transcriptional elements to regulate gene expression (Figure 4B). We next analysed the compartment strength. Notably, the Stra8‐positive germ cells had a significantly higher compartment strength than that of FGSCs (Figure 4C), which suggested the gradual compartmentalization from the undifferentiated stage to meiosis‐initiation stage of FGSCs. We next investigated the intrinsic properties of compartment A and B, such as gene numbers and GC content. Both stages of cells had higher gene numbers for compartment A than that of B, and Stra8‐positive germ cells had more active genes and significantly higher GC content than that of FGSCs (Figure 4D). These results indicated that FGSCs experienced chromatin compartment changes from the undifferentiated stage to meiosis‐initiation stage.

### 3.5 FGSCs underwent TAD changes from the undifferentiated stage to meiosis‐initiation stage

**FIGURE 5:**
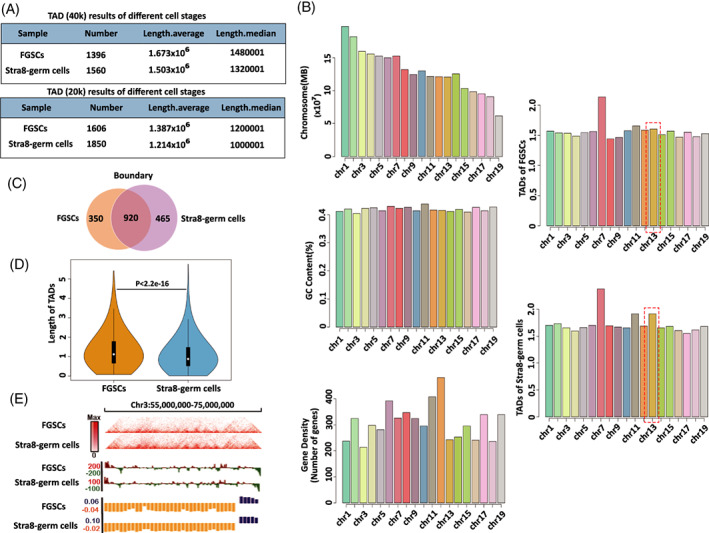
Analysis of TAD changes between FGSCs and Stra8‐positive germ cells. (A) Summary of TADs for cells at the two stages in 40 kb and 20 kb. (B) TAD description in each chromosome. Chromosome length, gene density, GC content and TAD density of each chromosome are shown. (C) Overlap of TAD boundaries between cells at the two stages. (D). Violin plot showing TAD size distribution cells at the two stages. White dot in the box indicated the median TAD size. (E) A typical meiosis‐associated region at Chr.3 described different TAD characteristics for cells at the two stages

Next, we examined the presence of TADs in the two stages of cells using the directional index (DI) value. TADs of 1396 for FGSCs and 1560 TADs for Stra8‐positive germ cells were identified at a resolution of 40 kb; 1606 TADs for FGSCs and 1850 TADs for Stra8‐positive germ cells were identified at a resolution of 20 kb (Figure 5A). The Stra8‐positive germ cells had larger numbers and smaller size of TADs compared with those in FGSCs (*n* = 1502, median size 1.34 Mb in FGSCs; *n* = 1705, median size 1.16 Mb in Stra8‐positive germ cells) (Figure 5A). We then performed TAD description in each chromosome including chromosome length, gene density and GC content, which were shown in Figure 5B. Chromosome 13 had higher TAD density in Stra8‐positive germ cells than FGSCs (Figure 5B). Next, we calculated the TAD boundaries based on Spearman's correlation. We identified 1270 TAD boundaries for FGSCs and 1385 TAD boundaries for Stra8‐positive germ cells. 72.4% (920 out of 1270, FGSCs) and 66.4% (920 out of 1385, Stra8‐positive germ cells) of TAD boundaries were shared between the two stages of cells (Figure 5C). Violin plot showed that the size of TAD boundaries in Stra8‐positive germ cells was significantly smaller than that in FGSCs (Figure 5D). In addition, we identified that a region typically associated with meiosis at chromosome 3 had more TADs in Stra8‐positive germ cells than that in FGSCs, and Stra8‐positive germ cells possessed weaker TADs than FGSCs at the region (Figure 5E). These results indicated that FGSCs underwent TAD changes from the undifferentiated stage to meiosis‐initiation stage.

### 3.6 chromatin loop analysis revealed that STRA8 regulated Trip13 transcription

**FIGURE 6:**
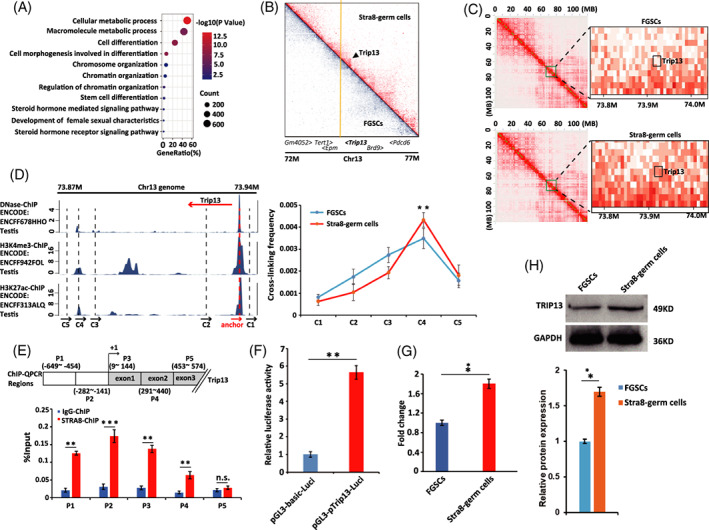
Analysis and verification of chromatin loop changes between FGSCs and Stra8‐positive germ cells. (A) GO enrichment analysis of chromatin loop changes according to Hi‐C sequencing in FGSCs and Stra8‐positive germ cells. (B) Heat map showed the chromatin loop changes at the Trip13 promoter region of Chr.13 in cells at the two stages. (C) An enlarged heat map of the chromatin loop changes at the Trip13 promoter region. The Stra8‐positive germ cells had more chromatin loops than FGSCs at the Trip13 gene region. (D) 3C assay measuring the crosslinking frequency in cells at the two stages. The Trip13 promoter (anchor) and five regions associated with complementary primers (C1 to C5) were amplified and tested by 3C‐qPCR. *p < 0.05, **p < 0.01, ***p < 0.001. (E) STRA8 ChIP‐qPCR was used to verify the interaction between STRA8 and the Trip13 promoter. IgG was used as a negative control. The locations of primers (P1 to P5) at the Trip13 promoter region were shown. *p < 0.05, **p < 0.01, ***p < 0.001, n.s. not significant. (F) Luciferase activity to evaluate STRA8 protein regulation of the Trip13 promoter. The pGL3‐Trip13‐Luc, pRL‐TK and PCDNA3.1‐Stra8 vectors were transfected into 3 T3 cells for 48 h. *p < 0.05, **p < 0.01, ***p < 0.001. (G) qRT‐PCR detected Trip13 gene expression in FGSCs and Stra8‐positive germ cells. *p < 0.05, **p < 0.01, ***p < 0.001. (H) Western blot analysis of TRIP13 in FGSCs and Stra8‐positive germ cells (left). Statistical results of western blotting (right). *p < 0.05, **p < 0.01, ***p < 0.001

To further investigate whether chromatin loops participate in the meiosis‐initiation processes through bringing distal regulatory elements into spatial proximity, we analysed the chromatin loop changes between FGSCs and Stra8‐positive germ cells. GO enrichment analysis of chromatin loop showed that genes were related to “cell differentiation,” “chromatin organization” and “development of female sexual characteristics” (Figure 6A). According to the term “cell differentiation,” we selected Trip13, which was required for recombination and higher‐order chromosome structures during meiosis.^36^ As shown in Figure 6B, the genome region of Trip13 had a higher strength of chromatin loops in Stra8‐positive germ cells than that in FGSCs. A higher resolution heat‐map showed the same result (Figure 6C).

To determine whether Stra8‐positive germ cells had contacts between enhancers and promoters to activate Trip13 transcription, we performed chromosome conformation capture (3C) to evaluate the relative frequencies of the Trip13 promoter interacting with the flanking enhancers or nearby control sites, according to the previously published high‐throughput sequencing data of mice testis (the promoter marker (H3K4me3) and enhancer markers (H3K27ac and DNase)) (Figure 6D).^37^ In Stra8‐positive germ cells, 3C revealed a frequent interaction between the Trip13 promoter and its distal enhancer, which were located about 57 kb apart (Figure 6D). This result confirmed that FGSCs underwent higher‐order chromatin loop changes at the genome region of Trip13 from the undifferentiated stage to the meiosis‐initiation stage.

To confirm that chromatin loop changes at the genome region of Trip13 play a pivotal role in meiosis initiation and further explore whether STRA8 regulated Trip13 transcription, we performed STRA8 ChIP‐qPCR. As shown in Figure 6E, STRA8 protein was found to bind the promoter region of mouse Trip13 (Figure 6E). We also performed dual‐luciferase reporter assays using a luciferase reporter vector containing 1.3 kb of the Trip13 core promoter and found that STRA8 expression increased activity of the reporter driven by the Trip13 promoter (Figure 6F). RT‐qPCR experiments showed that the expression of Trip13 gene was higher in Stra8‐positive germ cells than that in FGSCs (Figure 6G). Western blot also showed that Trip13 expression was higher in Stra8‐positive germ cells than that in FGSCs (Figure 6H).

Taken together, our results indicated that the chromatin loop at the genome region of Trip13 was established at early differentiation of FGSCs in vitro. The Trip13 promoter interacted with the distal enhancer to facilitate the access of STRA8 protein, further promoting Trip13 gene expression to participate the meiosis processes.

## 4 DISCUSSION

In our previous study,^10^ we optimized a variety of in vitro differentiation conditions to differentiate FGSCs into GV stage. However, the regulation mechanism was unclear, especially at the initiation of the meiotic cell cycle. In the present research, we constructed a dual fluorescence reporter system (Stra8‐EGFP, CMV‐mCherry) to trace the differentiated FGSCs. In FGSCs differentiated for 3 days in vitro, the ratio of STRA8‐positive germ cells to total cells reached the highest at day 3 (34.13%). Comparing STRA8‐positive germ cells with FGSCs, the average diameter increased from 13 μm to 16.8 μm. The phenotypic changes inevitably led to changes of transcription. RNA‐seq of the two stages confirmed 4788 differentially expressed genes, and 97% of upregulated genes in Stra8‐positive cells were also expressed in FGSCs, which is consistent with the conclusion that transcriptional up‐regulation at meiotic initiation mostly amplified gene expression.^19^ Furthermore, GO analysis revealed the enrichment of a series of meiosis‐associated and chromatin structure‐associated processes, which indicated that these processes and genes may be involved in meiotic initiation of FGSCs.

In mammals, chromatin conformation is highly organized, and chromatin undergoes drastic reorganization during cell processes. Recent years, with the development of chromosome conformation capture‐based technologies, studies have demonstrated the importance of chromatin structure in cell development.^38^, ^39^, ^40^, ^41^, ^42^, ^43^, ^44^ However, these reports have mainly focused on early embryonic development and spermatogenesis, and little attention has been paid to the meiosis‐initiation stage, especially for FGSC in vitro differentiation. Here, we examined FGSCs for the study of meiotic‐initiation stage, and using Hi‐C technology, we compared the global chromatin structure of Stra8‐positive germ cells with that of undifferentiated FGSCs. Contact heat maps showed that the chromatin structure was globally conserved with a distinct chromatin remodelling from the undifferentiated status to meiosis‐initiation stage, and a significantly smaller *cis/trans* ratio was observed for Stra8‐positive germ cells compared with that for FGSCs.

The genome is divided into A and B compartments.^45^ A compartments are associated with active (actively transcribed, open, GC‐rich, accessible) chromatin, and the B compartments are associated with inactive (inactively transcribed, closed, GC‐poor, less accessible) chromatin.^45^ In our analysis of A/B compartments in meiosis initiation in FGSCs, the B‐B compartments were increased and A‐A compartments were decreased from the undifferentiated stage to meiosis‐initiation stage in FGSCs. The A and B compartments were equally divided in FGSCs but Stra8‐positive germ cells underwent spatial plasticity in the rearrangement of compartment A and B. The global switch rate from FGSCs to Stra8‐positive germ cells was 29.81%, with 17.94% of compartment B (FGSCs) to compartment A (Stra8‐positive germ cells) and 11.87% of compartment A (FGSCs) to B (Stra8‐positive germ cells). These results indicated that chromatin compaction was gradually opening, allowing access for transcriptional elements to regulate gene expression. The compartment strength of Stra8‐positive germ cells was significantly higher than that of FGSCs, suggesting the gradual compartmentalization at the meiosis‐initiation stage. In analysing the gene numbers of compartments, Stra8‐positive germ cells had more active genes and GC content in compartment A than FGSCs.

Because the analysis of compartment was at the megabase scale, we further investigated TADs, which are large self‐interacting domains and fundamental structural units.^46^, ^47^, ^48^ The Stra8‐positive germ cells had larger numbers and smaller size of TADs compared with those in FGSCs (*n* = 1502, median size 1.34 Mb in FGSCs; n = 1705, median size 1.16 Mb in Stra8‐positive germ cells), especially for the chromosome 13. In mammals, TAD‐TAD are demarcated by boundaries and flanked by CTCF sites.^49^, ^50^, ^51^, ^52^, ^53^ Our results showed that 72.4% (920 out of 1270) of TAD boundaries for FGSCs was shared with 66.4% (920 out of 1385) of TAD boundaries for Stra8‐positive germ cells. Although most were conserved, 29.8% changed from the undifferentiated to meiosis‐initiation stage. A region typically associated with meiosis confirmed the results that Stra8‐positive germ cells had more but weaker TADs than FGSCs. These results indicated that FGSCs underwent TAD changes from the undifferentiated stage to meiosis‐initiation stage.

Within the TADs, regulatory interactions between promoter and enhancers are mediated through chromatin loops. Structural proteins (e.g. CTCF) play central roles in chromatin loop organization.^54^ Hence, we analysed the chromatin loop changes between the two stages and GO enrichment analysis confirmed that chromatin loop changes were associated with “cell differentiation,” “chromatin organization” and “development of female sexual characteristics.” From the term “cell differentiation,” we selected the Trip13 gene, because the genome region of this gene had much more strength of chromatin loops in Stra8‐positive germ cells than that in FGSCs. We performed chromosome conformation capture experiments and demonstrated that the Trip13 promoter contacted the distal enhancer (about 57 kb in distance), which indicated that from the undifferentiated stage to meiosis‐initiation stage, FGSCs experienced higher‐order chromatin loop changes at the genome region of Trip13. To confirm that chromatin loop changes at the genome region of Trip13 were involved in meiosis initiation and further explored the regulation mechanism of STRA8 and Trip13 gene, we performed STRA8 ChIP‐qPCR and constructed a Trip13 core promoter luciferase reporter. The results confirmed that STRA8 bound to Trip13 promoter to promote gene expression. We then examined the expression levels of Trip13 in Stra8‐positive germ cells and FGSCs. qRT‐PCR and western blot showed that mRNA and protein expression of Trip13 were higher in Stra8‐positive germ cells than levels in FGSCs. The above results indicated that at the stage of meiosis initiation, the Trip13 promoter contacted the distal enhancer to facilitate the access of STRA8 protein, further promoting Trip13 gene expression to participate the meiosis processes.

We analysed the 3‐D chromatin structure and found that chromatin loop at the genome region of Trip13 played a pivotal role in meiosis initiation. However, much work need to be done in future studies. First, during the differentiation process, whether spatial chromatin structure is altered by knocking out Trip13 in FGSCs and the target gene of Trip13 should be further explored. Second, our RNA‐seq data indicated that hormone‐associated processes were important for meiosis initiation, and future studies should examine the hormone‐associated genes that may be involved in regulation of 3‐D chromatin structure. Finally, our results showed that over 30% of TADs were specified in Stra8‐positive germ cells compared with those in FGSCs. How they are involved in meiosis? The alterations during development from the preleptotene stage to later stages should be further investigated.

In conclusion, our study revealed that when FGSCs were induced to differentiate into meiosis‐initiation stage, the cells underwent extensive transcriptome changes and chromatin structure remodelling. At the stage of Stra8‐positive germ cells, STRA8 was expressed. Furthermore, STRA8 bound to the Trip13 promoter which contacted distal enhancer to form spatial chromatin loop and promote its expression. Our findings on the regulation of chromatin structure deepen our understanding of early in vitro differentiation in FGSCs, which was important for the clinical treatment of female infertility.

## AUTHOR CONTRIBUTIONS

Yabin Zhang performed most of the experiments. Geng G Tian collected data and performed the RNA‐seq and Hi‐C analysis. Xiang Wang performed the RNA‐seq control of FGSCs. Changliang Hou conducted the pLVX‐mcherry‐Stra8‐EGFP dual luciferase reporter vector. Ji Wu designed the experiments conceptualization and supervized the project. Ji Wu and Xiaopeng Hu designed the experiments. Ji Wu and Xiaopeng Hu revised the final manuscript. All authors reviewed and approved the final paper.

## CONFLICT OF INTEREST

The authors declare that there is no conflict of interest regarding the publication of this article.

## Supporting information


**FIGURE S1** Immunofluorescence analysis of FGSCs with antibodies against MVH and OCT4. Scale bars: 20 μm

**FIGURE S2** Observed/expected number of contacts between the two stages of cells. The upper panel showed the observed/expected number of contacts between any pair of 19 euchromosomes in FGSCs or Stra8‐positive germ cells; the lower panel showed the chromosome length (Mb) of each chromosome

**TABLE S1** Sequences of the primers used in this paper

Supinfo

## Data Availability

All data needed to evaluate the conclusions in the paper are present in the paper and/or Supporting Information. Raw sequence data of Hi‐C and RNA‐seq have been submitted to the NCBI sequence read archive under accession number GSE195971. Additional data related to this paper may be requested from the authors.
