# Supplementary material for: Retinoic acid induced meiosis initiation in female germline stem cells by remodelling three‐dimensional chromatin structure
Source: Cell Prolif. 2022 May 28;55(7):e13242. doi: 10.1111/cpr.13242 (PMC9251051; doi:10.1111/cpr.13242)
Supplement: Supplementary file 3 — TABLE S1 Sequences of the primers used in this paper [file CPR-55-e13242-s001.docx]

Table S1: Sequences of the primers used in this paper.

| 1. Sequences of the primers used for RT-PCR. | |
| --- | --- |
| **Transcrpit** | **Sequence** |
| *Oct4* | F: AGCTGCTGAAGCAGAAGAGG |
|  | R: GGTTCTCATTGTTGTCGGCT |
| *Mvh* | F: GCCGTGGAGGATTTGGTCTA |
|  | R: AAGTGTCACCATTGCCTGAA |
| *Fragilis* | F: GTTATCACCATTGTTAGTGTCATC |
|  | R: AATGAGTGTTACACCTGCGTG |
| *Stra8* | F: TGTCGAAGGTGCATGGTTCA |
|  | R: TGAAGAGCCCTACCAGGGTG |
| *Sycp3* | F: ATGATGGAAACTCAGCAGCA |
|  | R: GCATGCCTCTTAGCTAATGTTTT |
| *Gapdh* | F: TCAGGAGAGTGTTTCCTCGTC |
|  | R: ACAAGCTTCCCATTCTCGGC |
| F, forward primer; R, reverse primer | |

| 2. Sequences of the primers used for qRT-PCR. | |
| --- | --- |
| **Transcrpit** | **Sequence** |
| *Stra8* | F: TGTCGAAGGTGCATGGTTCA |
|  | R: TGAAGAGCCCTACCAGGGTG |
| *ERa* | F: TCTGCCAAGGAGACTCGCTACT |
|  | R: GGTGCATTGGTTTGTAGCTGGAC |
| *Ccne1* | F: AAGCCCTCTGACCATTGTGTCC |
|  | R: CTAAGCAGCCAACATCCAGGAC |
| *Carm1* | F: TCGAGAGCTACCTCCATGCCAA |
|  | R: GGCTTTGGTGAACTGCTCCATG |
| *Trip13* | F: GTGGACAGCAACCTCATCACCT |
|  | R: TGCTCGACAGTCTGATGGTCAG |
| F, forward primer; R, reverse primer | |

| 3. Sequences of the primers used for STRA8 ChIP-qPCR. | |
| --- | --- |
| **Transcrpit** | **Sequence** |
| P1 | F: ACCCAGGACCCCAATTCCTT |
|  | R: CGTGAGAAGGAGGAGCTTA |
| P2 | F: CGGCTGCCGGTAGCTCGACCT |
|  | R: GCTCGGCGCTTTGCAAC |
| P3 | F: GGCGGTGGGCGACCTGAAGC |
|  | R: GGGGTCGGATCAGGTCGCG |
| P4 | F: CACTTCAGTTGACCCTAACA |
|  | R: CAGTTGAGAGCTGTGGGGTCCT |
| P5 | F: AGCACAGCCTAACGATGAGCT |
|  | R: TGGCTTTGCAAATTAAATACT |
| F, forward primer; R, reverse primer | |

| 4. Sequences of the primers used for Trip13 luciferase activity analysis. | |
| --- | --- |
| **Transcrpit** | **Sequence** |
| *Trip13-promoter* | F: cgtgctagcccgggctcgagtttccgtttcttctcttcct |
|  | R: agtaccggaatgccaagcttcttctccgatgtaggaggat |
| F, forward primer; R, reverse primer | |

| 5. Sequences of the primers used for 3C. | |
| --- | --- |
| **Transcrpit** | **Sequence** |
| Anchor | TTCTCACCGGATCCCGTGCTAT |
| C1 | GTGTAGAAGTTTAGCATTATCTG |
| C2 | GATCTTAGCCATTCTGAGTGGTG |
| C3 | CTTCCTCTCTCCCTTCCTGTGACT |
| C4 | AGTGTTCTACACTACACAGTGTG |
| C5 | TAATCAGGGCAGCAGGGGCAAA |
