# Supplementary material for: Retinoic acid induced meiosis initiation in female germline stem cells by remodelling three‐dimensional chromatin structure
Source: Cell Prolif. 2022 May 28;55(7):e13242. doi: 10.1111/cpr.13242 (PMC9251051; doi:10.1111/cpr.13242)
Supplement: Supplementary file 4 — Supinfo [file CPR-55-e13242-s004.docx]

**Supplemental information**

**Retinoic acid induced meiosis initiation of female germline stem cells by remodeling three-dimensional chromatin structure**

Yabin Zhang^#^, Geng G Tian^#^, Xiang Wang, Changliang Hou, XiaopengHu^*^, Ji Wu^*^

^
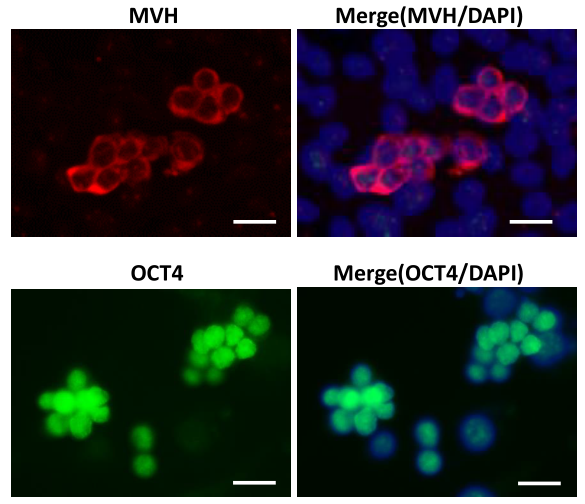
^

**Supplemental Figure 1** Immunofluorescence analysis of FGSCs with antibodies against MVH and OCT4. Scale bars: 20 μm.

.


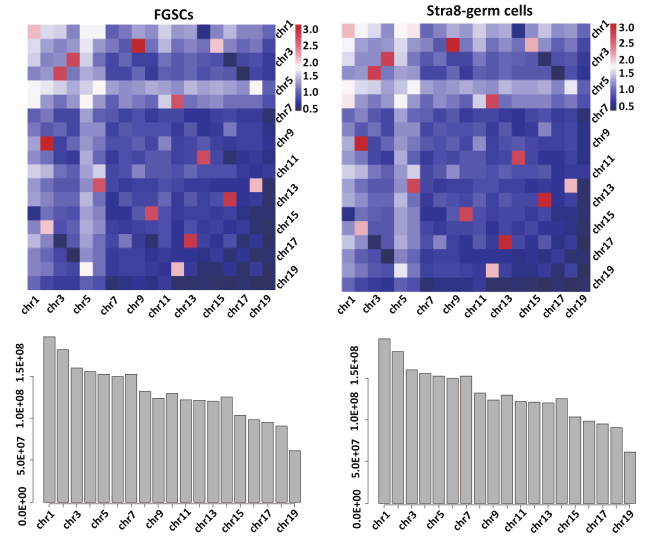


**Supplemental Figure 2** Observed/expected number of contacts between the two stages of cells.

The upper panel showed the observed/expected number of contacts between any pair of 19 euchromosomes in FGSCs or Stra8-positive germ cells; the lower panel showed the chromosome length (Mb) of each chromosome.

**Supplemental materials and Methods**

**2.1 Animals**

Pregnant  C57BL/6 female mice at 12.5dpc were purchased from SLAC Laboratory Animal Co., Ltd (Shanghai, China). All animal procedures were approved by the Institutional Animal Care and Use Committee of Shanghai Jiao Tong University and performed in accordance with the National Research Council Guide for the Care and Use of Laboratory Animals.

**2.2 FGSC culture**

For FGSC culture, in brief, FGSCs were cultured on mitotically inactivated SIM mouse embryo-derived thioguanine-and ouabain-resistant (STO; ATCC, Manassas, VA, USA) feeders in minimum essential medium alpha (MEMα; Invitrogen, Carlsbad, CA, USA), which was supplemented with 10% fetal bovine serum (FBS; Life Technologies, Carlsbad, CA, USA), 10 ng/mL mouse leukemia inhibitory factor (mLif; Santa Cruz Biotechnology, CA, USA), 20 ng/mL mouse epidermal growth factor (EGF; PeproTech, NJ, USA), 10 ng/mL basic fibroblast growth factor (bFGF; BD Biosciences, Franklin Lakes, NJ, USA), 10 ng/mL mouse glial cell line-derived neurotrophic factor (GDNF; R&D Systems, Minneapolis, MN, USA), 1 mM nonessential amino acids (NEAA; Invitrogen Life Sciences, MA, USA), 2 mM L-glutamine (Amresco, Radnor, PA, USA), 1 mM sodium pyruvate (Amresco) and 0.1 mM β-mercaptoethanol (Sigma-Aldrich, St. Louis, MO, USA). FGSCs were passaged every 4–7 days.

**2.3 Immunofluorescence staining**

FGSCs were gently washed with phosphate buffered saline (PBS) and fixed with 4% paraformaldehyde at room temperature for 15–20 min. After two washes with PBS, cells were blocked with 20% goat serum for 30 min and treated with 0.5% Triton-X 100 in a humidified box at 37°C for 15 min. Cells were then incubated with primary antibodies against STRA8 (1:100; OriGene, Rockville, MD, USA) in a humidified box overnight at 4°C. Cells were washed with PBS and then incubated with PBS-diluted secondary antibody labelled with TRITC (1:150; goat anti-rabbit IgG; ProteinTech) for 60 min away from light. Nuclei were stained with DAPI. Images were acquired by a fluorescence microscope (Leica, Wetzlar, Germany).

**2.4 Vector construction**

For construction of the pLVX-mcherry-Stra8-EGFP dual luciferase reporter vector, we cloned the 1.4 kb Stra8 promoter and EGFP sequences by PCR as described in a previous report.^1^ We inserted the sequences into the pLVX-mCherry-N1 basic vector at SanDI and ClaI sites. For the PCDNA3.1-Stra8 vector, we cloned the 1182 bp mouse Stra8 coding sequence into the PCDNA3.1 vector at BamHI and XhoI. For the pGL3-mTrip13 promoter dual luciferase reporter vector, we cloned the approximate 1.3 kb mTrip13 promoter sequence into the pGL3-basic vector(Sigma-Aldrich) by homologous recombination at XhoI and HindIII. All primers are shown in Table S1.

**2.5 Lentivirus infection**

The lentivirus of pLVX-mCherry-Stra8-EGFP dual fluorescent reporter vector was packaged by and purchased from HanBio Inc. (Shanghai, China). FGSCs were plated in 24-well plates until they reached approximately 50% confluence and then incubated with a 1:1 mixture of culture medium and lentivirus (titer: 2×10^8^) for 16 h. To obtain stable clones, the infected FGSCs were screened by puromycin (5 µg/mL) for 4 days.

**2.6 Western blot analysis**

Cells were lysed in radioimmunoprecipitation assay buffer (RIPA; Beyotime) for 30 min and lysates were centrifuged at 12,000×g for 30 min. Equal amounts of protein were separated on 12% SDS-PAGE gels and transferred onto a polyvinyl difluoride (PVDF) membrane. The membrane was blocked with 5% non-fat milk in Tris-buffer saline (TBS)-T (TBS with 0.05% Tween-20) for 2 h with gentle shaking at room temperature. Membranes were incubated at 4°C overnight with the following primary antibodies: anti-STRA8 (rabbit-anti-Stra8, 1:500, OriGene), anti-GAPDH (mouse-anti-gapdh, 1:8000, Abcam) and anti-mTrip13 (mouse-anti-Trip13,1:500, Santa Cruz Biotechnology). The membranes were washed three times with TBST buffer and incubated with secondary antibodies (Proteintech) for 1 h at room temperature. Protein bands were visualized using ECL reagent (Beyotime) and scanned with a Tanon 4600SF (Tanon, Shanghai, China). The density of protein bands was quantified with Image J software.

**2.7 Reverse transcription PCR (RT-PCR) and quantitative real-time PCR (qRT-PCR)**

Total RNA was extracted with Trizol reagent (Life Technologies). cDNA (1000 ng) was synthesized by using a reverse transcription kit (Takara, Tokyo, Japan). RT-PCR analysis was carried out with Taq DNA polymerase(Takara). qRT-PCR was performed using SYBR mix in an Applied Biosystems® 7500 Real-Time PCR System in accordance with the manufacturer’s instructions (Applied Biosystems, Foster City, CA, USA). Transcript levels were normalized to Gapdh level. The 2^-ΔΔCt^ method was used to analyze data. All primers are shown in Table S1.

**2.8 Gene Ontology enrichment analysis**

Gene Ontology enrichment analyses were performed to identify the biochemical processes of differentially expressed mRNAs. The data of differentially expressed mRNAs were uploaded to DAVID (http://david.abcc.ncifcrf.gov/home.jsp). Fisher’s exact test was used to identify the significant results; the false discovery rate was applied to correct the P values (P < 0.05; fold change > 1.5).

**2.9 Hi-C data processing and mapping**

Low-quality reads were filtered with BBmap (version 38.16). The high quality paired-end Hi-C reads were mapped, processed and bias corrected using HiCPro (version 2.7).^2^  Next, the reads were aligned to the mouse reference genome (mm9) using the bowtie2 algorithm end-to-end algorithm (“very-sensitive” option) .^3^ Fragments of invalid self-ligated, uncut DNA reads and PCR artefacts were discarded. We divided the genome into bins of equal size and the valid read pairs were then binned at a specific resolution. Next, we applied the approach of ICE for Hi-C normalization^4^ to correct biases in the raw matrix, such as GC content, mappability and effective fragment length in Hi-C data. The contact matrices were finally generated at 20-kbp bin size resolutions.

**2.10 Contact probability P(s) analysis**

We calculated the P(s) with normalized interaction matrices in 20-kb resolution, as described previously^5^. In brief, we first divided the genome into 20-kb logarithmically spaced bins and counted the number of interactions at corresponding distances for each bin (separated by 40, 80, 120 and 160 kb). To obtain the probability P(s), we then divided the number of interactions in each bin by the total number of possible region reads. Finally, the sum of P(s) was further normalized over the range of the distances as 1.

**2.11 Identification of compartments A and B**

HiTC (from R package) was applied to generate the PC1 and calculate compartments A and B using 400-kb normalized matrices with PCA^6^. The options were as follows: normPerExpected = TRUE, npc = 1, for which a positive Spearman’s correlation PC1 value indicated the A compartment, while a negative PC1 value indicated the B compartment. To investigate the compartment switching of each chromosome, we defined switched bins only if PC1 eigenvectors changed in the same direction for two replicates.

**2.12 TAD calling, TAD boundaries and TAD types**

The location of the TADs was identified using the directional index (DI) value, as previously reported^7^. We calculated the DI value at 20-kb resolution based on the ICE-normalized matrix and used the hidden Markov model to predict the states of DI for final TAD generation.

The TAD boundaries (distance between two adjacent TADs) were defined as those <400 kb. Regarding the TAD types, if the boundaries regions were located within 10× bins (200 kb), they were considered as ‘common,’ while boundaries located at >200 kb were considered as ‘cell-type-specific.’

**References**

1. Nayernia K, Li M, Jaroszynski L, Khusainov R, Wulf G, Schwandt I, Korabiowska M, Michelmann HW, Meinhardt A, Engel W (2004) Stem cell based therapeutical approach of male infertility by teratocarcinoma derived germ cells. Hum Mol Genet 13(14):1451–1460. doi:[10.1093/hmg/ddh166ddh166](https://doi.org/10.1093/hmg/ddh166ddh166)

2. Servant N, Varoquaux N, Lajoie BR, Viara E, Chen CJ, Vert JP, Heard E, Dekker J, Barillot E. HiC-Pro: an optimized and flexible pipeline for Hi-C data processing. Genome Biol. 2015 Dec 1;16:259

3. Langmead B, Salzberg SL. Fast gapped-read alignment with Bowtie 2. Nat Methods. 2012 Mar 4;9(4):357-9. doi: 10.1038/nmeth.192

4. Imakaev M, Fudenberg G, McCord RP, Naumova N, Goloborodko A, Lajoie BR, Dekker J, Mirny LA. Iterative correction of Hi-C data reveals hallmarks of chromosome organization. Nat Methods. 2012 Oct;9(10):999-1003

5. Naumova N, Imakaev M, Fudenberg G, Zhan Y, Lajoie BR, Mirny LA, Dekker J. Organization of the mitotic chromosome. Science. 2013 Nov 22;342(6161):948-53. doi: 10.1126/science.1236083

6. Servant N, Lajoie BR, Nora EP, Giorgetti L, Chen CJ, Heard E, Dekker J, Barillot E. HiTC: exploration of high-throughput 'C' experiments. Bioinformatics. 2012 Nov 1;28(21):2843-4

7. Dixon JR, Selvaraj S, Yue F, Kim A, Li Y, Shen Y, Hu M, Liu JS, Ren B. Topological domains in mammalian genomes identified by analysis of chromatin interactions. Nature. 2012 Apr 11;485(7398):376-80
